# *In Vitro* and *In Vivo* Antitumor Effects of n-Butanol Extracts of *Pterocephalus hookeri* on Hep3B Cancer Cell

**DOI:** 10.1155/2015/159132

**Published:** 2015-05-19

**Authors:** Chenxu Guo, Yingchun Wu, Yuanzhang Zhu, Yanchun Wang, Lili Tian, Yi Lu, Cong Han, Guofu Zhu

**Affiliations:** ^1^School of Pharmacy, Shanghai University of Traditional Chinese Medicine, Shanghai 201203, China; ^2^Department of Traditional Chinese Medicine, Henan Province People's Hospital, Zhengzhou 221000, China

## Abstract

*Pterocephalus hookeri* is a widely applied Tibetan medicinal prescription for treatment of diseases such as flu, rheumatoid arthritis, and enteritis in China. It has been reported that* Pterocephalus hookeri *has anti-inflammatory and analgesic actions. However, the antitumor activity of* Pterocephalus hookeri *remains unknown. In the present study, we demonstrate that n-butanol extracts of* Pterocephalus hookeri* (YSC-ZDC) has a strong antitumor activity against hepatoma carcinoma cell* in vitro* and* in vivo*. YSC-ZDC inhibited proliferation of all cancer cell lines and significantly inhibited Hep3B cells proliferation in a dose- and time-dependant manner. Transmission electron microscopy, hoechst 33258 staining, and flow cytometry analysis revealed that YSC-ZDC induced apoptosis in Hep3B cells. YSC-ZDC treatment dramatically inhibited PDK1 and Akt phosphorylation in Hep3B cells. Moreover, YSC-ZDC increased Bax expression and inhibited Bcl-2 expression. In addition, YSC-ZDC inhibited growth hepatoma xenografts* in vivo *with no effect on body weight and spleen index. Consistent with results* in vitro*, YSC-ZDC increased Bax expression and inhibited Bcl-2 expression in tumor tissue. Taken together, this study shows YSC-ZDC with an antitumor activity both * in vitro* and *in vivo*. Its mechanism underlying is related to blocking of the Akt pathway and regulation of Bcl-2 family proteins expression.

## 1. Introduction

Hepatocellular carcinoma (HCC) is the fifth most common malignancy worldwide and the third leading cause of mortality in all cancer-related diseases with the highest incidence occurring in eastern and southeastern Asia, middle and western Africa, southern Europe, and South America [1]. Unfortunately, the overall response to liver cancer treatment is unsatisfactory, mainly due to late diagnosis and poor treatment efficacy; resistance to chemotherapeutic drugs; and metastasis to other organs which negatively affect the mortality rate [2]. Consequently, new and effective treatment options for liver cancer are urgently needed, supporting the fact that plant-derived natural products occupy an important position in the treatment of diseases due to their minimal side effects and lack of multidrug resistance [3, 4].

Tibetan herbs, as traditional Chinese herbal medicines, have been used to treat a wide variety of clinical diseases in China for several centuries. Numerous studies have identified that Tibetan herbs have proapoptotic and anticancer properties [5, 6].* Pterocephalus hookeri* is a popular Tibetan herb, locally known as “Bang-zi-du-wu” in the Tibetan language and recorded in the Tibetan medicine book as “Si-Bu-Yi-Dian” [7].* Pterocephalus hookeri *has multiple traditional uses in the treatment of illnesses such as cold, flu, rheumatoid arthritis, and enteritis in Tibet [8, 9]. Recently, it was reported that the ethanol and aqueous extracts of* Pterocephalus hookeri* have anti-inflammatory and analgesic actions [7]. However, the effect of* Pterocephalus hookeri* on antitumor activity remains unknown.

Our previous study revealed that four new bis-iridoids isolated from* Pterocephalus hookeri* inhibited TNF-*α*-induced NF-*κ*B-dependent promoter activity [8]. In this study, we investigated antitumor activities of n-butanol extract of* Pterocephalus hookeri* (YSC-ZDC). The results indicated that YSC-ZDC inhibited proliferation of different cancer cell lines, downregulated phosphorylation of PDK1, and regulated Bcl-2 family proteins expression. In addition, YSC-ZDC inhibited tumor growth in xenograft tumor mouse model, suggesting that YSC-ZDC may have use as a treatment for cancer.

## 2. Materials and Methods

### 2.1. Identification and Preparation of* Pterocephalus hookeri*

*Pterocephalus hookeri* specimens were collected in April, 2014, from Qinghai province in southwest China. The taxonomical identification of the plants was authenticated by Professor Zhaozhi Li, School of Pharmacy, Shanghai University of TCM, where a voucher specimen (YSC-20120415) has been deposited. Pulverized* Pterocephalus hookeri* (800 g) was extracted (three times) with 80% ethanol for 1 h. The recovered dried sample in aqueous solution (200 mL) was extracted with different organic solvents in the ratio of 1 : 1 to yield petroleum ether, chloroform, and water-saturated n-butanol extracts. The qualitative chemical profile (fingerprint) of n-butanol extract was analyzed by high-performance liquid chromatography (HPLC) as described in the Chinese Pharmacopoeia.

### 2.2. Cell Culture and Reagents

Hep3B, ECA-109, Caco-2, Hela, K562, MCF-7, and A549 cell lines were obtained from the Type Culture Collection of the Chinese Academy of Sciences (Shanghai, China) and cultured in Dulbecco's modified Eagle's medium (DMEM) and RPMI1640 medium (Thermo Scientific, Waltham, MA, USA) containing 10% fetal bovine serum (GIBCO, Grand Island, NY), 100 U/mL penicillin, and 100 U/mL streptomycin (Sigma, St. Louis, MO, USA) in a humidified atmosphere of 5% CO_2_ at 37°C. Anti-Bcl-2 (#2870), anti-Bax (#5023), anti-Bcl-xL (#2762), anti-Bad (#9292), anti-PI3K (#4225), anti-phospho-PI3K (#4228), anti-PDK1 (#3062), anti-phospho-PDK1 (#3438), anti-Akt (#9272), anti-phospho-Akt (#4058), and anti-GAPDH (#5174) antibodies were purchased from Cell Signaling Technology (Danvers, MA, USA). 3-(4, 5-dimethylthiazol-2-yl) 2, 5-diphenyltetrazolium bromide (MTT) were purchased from Life Technologies (Carlsbad, CA, USA). All other chemicals were obtained from Sigma-Aldrich (Saint Louis, MO, USA).

### 2.3. Cell Proliferation Assay

Cells (4 × 10^3^ cells/well) were cultured in 96-well plate and treated with YSC-ZDC at different concentrations for the indicated times. After treatment, cells were incubated with 10 *μ*L/well 5 mg/mL MTT for 4 h. MTT formazan production was dissolved by DMSO (200 *μ*L/well). MTT assay was performed to measure cell viability by a plate reader at 492 nm (BioTek, Winooski, VT, USA).

### 2.4. Hoechst 33258 Staining

Cells (2 × 10^5^) were cultured in 6-well plate and treated with 100 *μ*g/mL YSC-ZDC and 20 *μ*g/mL 5-fluorouracil (5-Fu). After 48 h treatment, cells were washed twice with phosphate-buffered saline (PBS) and fixed with 4% paraformaldehyde for 30 min. Following an additional PBS wash, cells were incubated in Hoechst 33258 solution (5 *μ*g/mL) for 10 min in the dark at 37°C. Cells were washed twice in PBS and morphological changes of nuclear were visualized and analyzed using an inverted fluorescence microscope (Olympus IX71, Tokyo, Japan).

### 2.5. Transmission Electron Microscopy

Cells (2 × 10^5^) were cultured in 6-well plates and treated with 100 *μ*g/mL YSC-ZDC and 20 *μ*g/mL 5-Fu. After 48 h treatment, cells were fixed with 2.5% glutaraldehyde for 24 h at 4°C and then postfixed in 1% osmium tetroxide for 2 h at room temperature. The samples were dehydrated, infiltrated, and embedded in conventional epoxy resin. Ultrathin sections were cut and mounted on 200-mesh copper grids. The sections were stained with uranyl acetate and lead citrate and then examined using transmission electron microscopy (TEM, Philips Tecnai-12, Amsterdam, Netherlands).

### 2.6. Flow Cytometry

Hep3B cells (2 × 10^5^/well) were cultured in 6-well plates and treated with YSC-ZDC at different concentrations. After treatment for 48 h, cells were collected and labeled with a fluorescein isothiocyanate (FITC) Annexin V Apoptosis Detection Kit (BD Biosciences). Apoptotic rates were determined by flow cytometry (BD Biosciences) and analyzed with Flowjo 7.6.1 software.

### 2.7. Western Blot

Briefly, cells (2 × 10^5^/well) were cultured in 6-well plate and treated with YSC-ZDC at different concentrations. After treatment, cells were harvested and lysed with lysis buffer containing 1 mM EDTA, 150 mM NaCl, 50 mM Tris-HCl (pH 7.5), protease and phosphatase inhibitor cocktails (Sigma). Tumor tissues were homogenized and lysed with lysis buffer. The protein concentrations were determined using the Bradford protein assay reagent (Bio-Rad, Hercules, CA, USA). For western blot analysis, equal amounts of total protein were mixed with SDS sample buffer and separated by SDS-polyacrylamide gel electrophoresis. After electrophoresis, protein was blotted on a PVDF membrane (Millipore, Billerica, MA, USA) and blocked for 1 h in 5% nonfat milk. The membrane was incubated with primary antibodies at 4°C overnight and Horseradish peroxidase- (HRP-) conjugated anti-rabbit IgG secondary antibody for 1 h. Protein bands were visualized using enhanced chemiluminescence reagents according to the manufacturer's instructions (Thermo Scientific). The densities of protein bands were determined by Image J software.

### 2.8. Real-Time Polymerase Chain Reaction

Total RNA from Hep3B cells was isolated using Trizol Reagent (Invitrogen, Carlsbad, CA) according to the manufacturer's instructions. The RNA was used for reverse transcription with M-MLV reverse transcriptase (TaKaRa Biotechnology, Japan). The SYBR green DNA PCR kit (TaKaRa Biotechnology, Japan) was used for real-time quantitative PCR analysis. The nucleotide sequences of the primers used in this study were as follows: Bcl-2: sense 5′-CGC CCT GTG GAT GAC TGA GTA C-3′, antisense 5′-GGG CCG TAC AGT TCC ACA AAG-3′; Bax: sense 5′-CCACCA AGA AGC TGA GCG AGT-3′, antisense 5′-TGC CAC TCG GAA AAA GAC CTC-3′; Bcl-xL: sense 5′-CAC TGT GCG TGG AAA GCG TA-3′, antisense 5′-AAA GTG TCC CAG CCG CC′; Bad: sense 5′-GGA AGA CGC TAG TGC TAC AG-3′, antisense 5′-GAG CCT CCT TTG CCC AAG TT-3′ and GAPDH: sense 5′-GCA CAA ACG AGG GGA GTA CAT CAA-3′, antisense 5′-CTC AGG AGT CTC CAC ATG GAA GGT-3′.

### 2.9. Xenograft Tumor Mouse Model

Athymic nude mice (5-week-old male BALB/c nu/nu) were obtained from Shanghai Laboratory Animal Center of Chinese Academy of Science (Shanghai, China) and housed in sterile filter-topped cages. Hep3B cells (10^6^ cells/200 *μ*L) were subcutaneously injected into the left flank of the mice. Tumor size was measured using a caliper at 2-day intervals, and the volume was calculated by the modified formula *V* = 1/2 (length × width^2^). After tumors were established (~30 mm^3^), different doses of YSC-ZDC were dissolved in 0.5% CMC-Na and administered via gavage daily for 20 days. The 5-FU was dissolved in 0.9% normal saline (NS) and administered intraperitoneally (20 mg/kg/day) for 2-day intervals as a positive control group. Negative control animals were injected with vehicle (0.5% CMC-Na) alone. After the 20-day treatment, mice were sacrificed and tumor tissues were collected for analysis. All animal experiments were approved by the Ethics Committee for Animal Experimentation, Shanghai University of Traditional Chinese Medicine.

### 2.10. Immunohistochemistry

Immunohistochemistry (IHC) was performed as previously reported [10]. Briefly, tumor tissues were rinsed with PBS, fixed in 4% paraformaldehyde, embedded in paraffin, and cut into sections. Tissue sections (5 *μ*m) were dewaxed, rehydrated, and treated with 3% hydrogen peroxide (H_2_O_2_) in methanol (v/v). After incubation in a blocking solution (5% bovine serum albumin) for 1 h, the slides were incubated with primary antibodies (Bcl-2 1 : 200, BD Biosciences; Bax, 1 : 200, ImmunoWay, Newark, DE, USA) overnight at 4°C in a humidified chamber and peroxidase-conjugated secondary antibodies (HRP-labeled, Jackson ImmunoResearch, West Grove, PA, USA). A positive reaction was visualized by incubating these slides with stable 3,3′-diaminobenzidine (DAB) and counterstaining with Mayer's hematoxylin. The images were visualized and analyzed using an inverted fluorescence microscope (Olympus IX71, Tokyo, Japan).

### 2.11. Statistical Analysis

Statistical significance was determined using Student's *t*-tests and one-way analysis of variance (ANOVA) with SPSS18.0 software (SPSS Inc., Chicago, IL, USA). *P* < 0.05 was considered statistically significant.

## 3. Results

### 3.1. HPLC Analysis of n-Butanol Extract of* P. hookeri* (YSC-ZDC) and Chemical Profile

**Figure 1:**
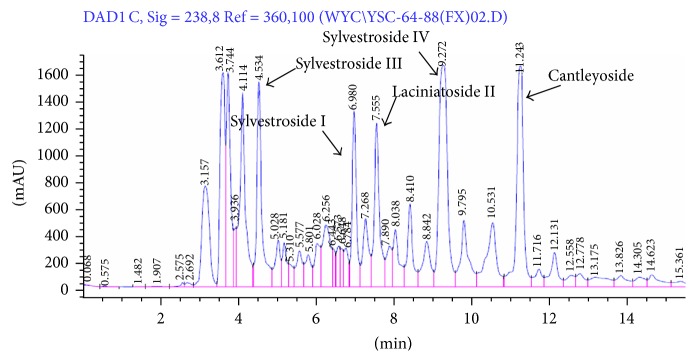
HPLC chromatogram of YSC-ZDC. Five compounds were deduced by comparing individual peak retention times with those of the standard substances.

The HPLC analysis of YSC-ZDC showed that over 15 peaks were detected within 15 min, among which 5 compounds were identified by comparing individual peak retention times with the standard substances (Figure 1). They were sylvestroside III (4.534 min), sylvestroside I (6.900 min), laciniatoside II (7.555 min), sylvestroside IV (9.272 min), and cantleyoside (11.243 min).

### 3.2. YSC-ZDC Inhibited Proliferation of Different Cancer Cell Lines

To determine the effects of YSC-ZDC on cell proliferation, we initially treated seven cancer cell lines including Hep3B, ECA-109, Caco-2, Hela, K562, MCF-7, and A549 from different tissues with YSC-ZDC at different concentrations for 48 h and the 50% inhibitory concentration (IC50) was calculated. As shown in Table 1, YSC-ZDC inhibited proliferation of all seven cancer cell lines. The IC50 values are around 90.8–318.2 *μ*g/mL. YSC-ZDC exhibited the strongest antiproliferation effect on Hep3B cell line with an IC50 of 90.8 *μ*g/mL.

### 3.3. YSC-ZDC Induced Apoptosis in Hep3B Cells

**Figure 2:**
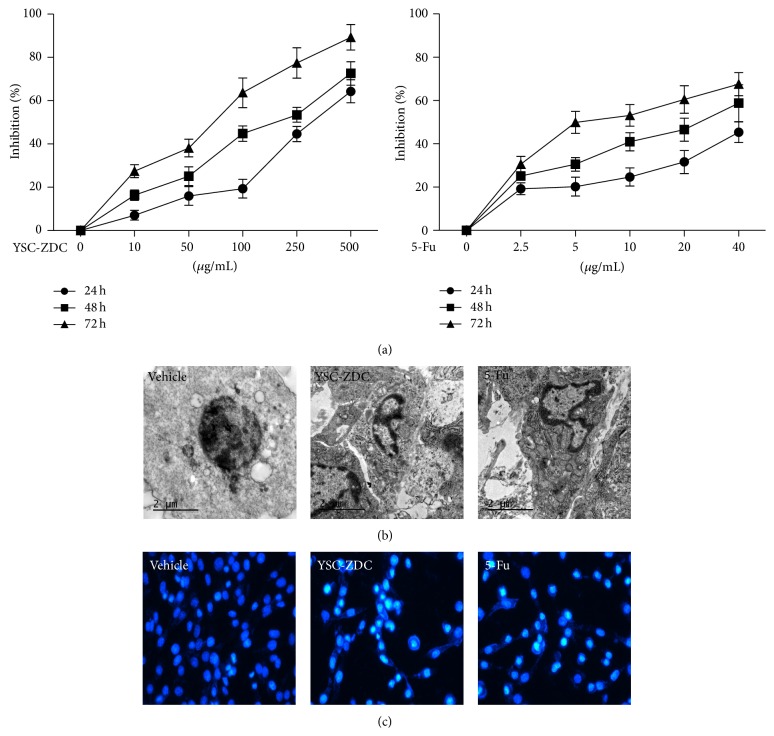
YSC-ZDC inhibited the proliferation of Hep3B cells. (a) YSC-ZDC inhibited cell proliferation in Hep3B cells. Cells were treated with YSC-ZDC (0–500 *μ*g/mL) or 5-Fu (0–40 *μ*g/mL) for the indicated times. Cell proliferation was determined by MTT assay. Data are shown as means ± S.E.M of three independent experiments. (b) Cells were treated with 100 *μ*g/mL YSC-ZDC or 20 *μ*g/mL 5-Fu for 48 h and then fixed with 2.5% glutaraldehyde and postfixed in 1% osmium tetroxide. Ultrathin sections were examined using transmission electron microscopy. (c) Cells were treated with 100 *μ*g/mL YSC-ZDC or 20 *μ*g/mL 5-Fu for 48 h and incubated with Hoechst 33258 (5 *μ*g/mL) for 10 min. Morphological changes of nuclear were visualized and analyzed using an inverted fluorescence microscope.

To evaluate the effects of YSC-ZDC on proliferation of Hep3B cells, Hep3B cells were treated with YSC-ZDC at different concentrations for 24, 48, and 72 h. As shown in Figure 2(a), YSC-ZDC significantly inhibited Hep3B cells proliferation not only in a dose-dependent but also in a time-dependent manner. A chemotherapeutic drug 5-Fu also inhibited Hep3B cells proliferation in a time- and dose-dependent manner. The majority of chemotherapeutic agents utilize the apoptotic pathway to induce cancer cell death [11]. To determine the effect of YSC-ZDC on apoptosis, the morphological changes in Hep3B cells were detected using transmission electron microscopy and Hoechst 33258 staining. As shown in Figures 2(b) and 2(c), no nucleus changes were found in vehicle-treated cells. After 100 *μ*g/mL YSC-ZDC treatment for 48 h, cell shrinkage and chromatin condensation were found in Hep3B cells. As a positive control, 20 *μ*g/mL 5-Fu-treated cells also exhibited chromatin condensation. These data indicated that YSC-ZDC markedly induced apoptotic cell death in Hep3B cells.

**Figure 3:**
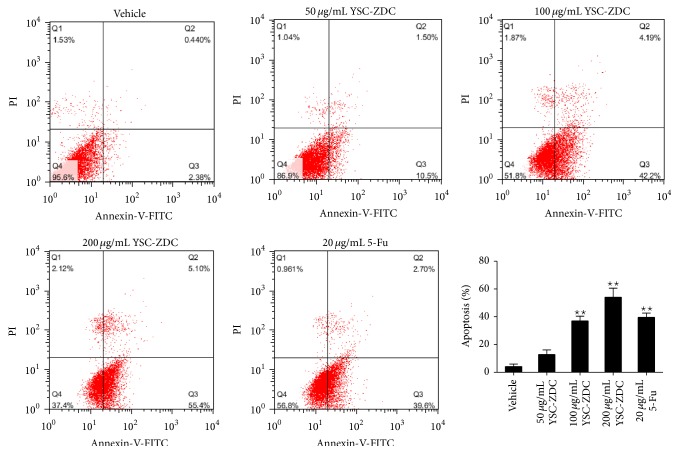
YSC-ZDC induced apoptosis in Hep3B cells. Cells were treated with YSC-ZDC at different concentrations or 20 *μ*g/mL 5-Fu for 48 h. Apoptotic cells were determined by Annexin V/PI staining using flow cytometry. Data are shown as means ± S.E.M of three independent experiments. ^*^ *P* < 0.05,^ **^ *P* < 0.01 versus vehicle.

In order to confirm the effect of YSC-ZDC on apoptosis, we used Annexin V/PI double staining to identify the involvement of apoptosis in YSC-ZDC-induced cell death. As shown in Figure 3, the flow cytometry result showed that early apoptotic and late apoptotic rates increased significantly up to 55.4% and 5.10%, respectively, after 200 *μ*g/mL YSC-ZDC treatment. YSC-ZDC dose-dependently induced apoptosis in Hep3B cells. These results indicated that YSC-ZDC inhibited proliferation and significantly induced apoptosis in Hep3B cells.

### 3.4. YSC-ZDC Inhibited PI3K/Akt Signaling in Hep3B Cells

**Figure 4:**
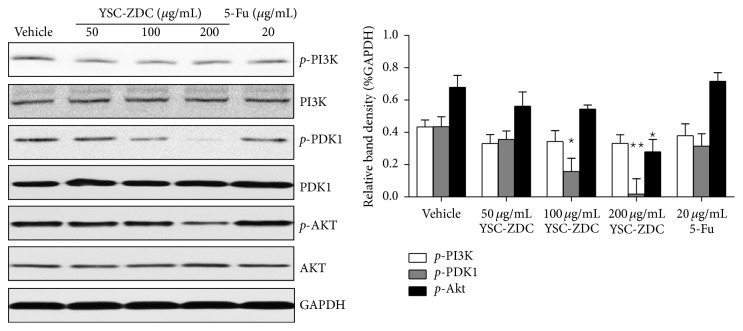
YSC-ZDC inhibited PI3K/AKT signaling pathway in Hep3B cells. Cells were treated with YSC-ZDC at different concentrations or 20 *μ*g/mL 5-Fu for 12 h. The levels of total and phosphorylated PI3K, PDK1, and Akt were determined by western blot. Bands were analyzed by densitometry. Data are shown as mean ± S.E.M of three independent experiments. ^*^ *P* < 0.05, ^**^ *P* < 0.01 versus vehicle.

The serine/threonine kinase AKT is a highly conserved central regulator of growth-promoting signals in multiple cell types, and inhibition of AKT activity leads to aberration of cell growth [12]. We examined the effect of YSC-ZDC on PI3K/Akt pathway in Hep3B cells. As shown in Figure 4, YSC-ZDC has no effect on PI3K phosphorylation. But YSC-ZDC treatment significantly inhibited the phosphorylation of PKD1 in a dose-dependent manner without any effect on total PDK1 expression. Moreover, as a downstream protein of PDK1, phosphorylation of Akt also was inhibited by YSC-ZDC treatment. However, 5-Fu exerts its anticancer effects through the inhibition of thymidylate synthase and the incorporation of its active metabolites into RNA and DNA. Western blot showed that 5-Fu has no effect on PI3K, PDK1, and Akt protein expression (Figure 4). These data indicated that PI3K/Akt pathway is involved in YSC-ZDC-induced apoptotic cell death.

### 3.5. The Effect of YSC-ZDC on Bcl-2 Family Proteins Expression in Hep3B Cells

**Figure 5:**
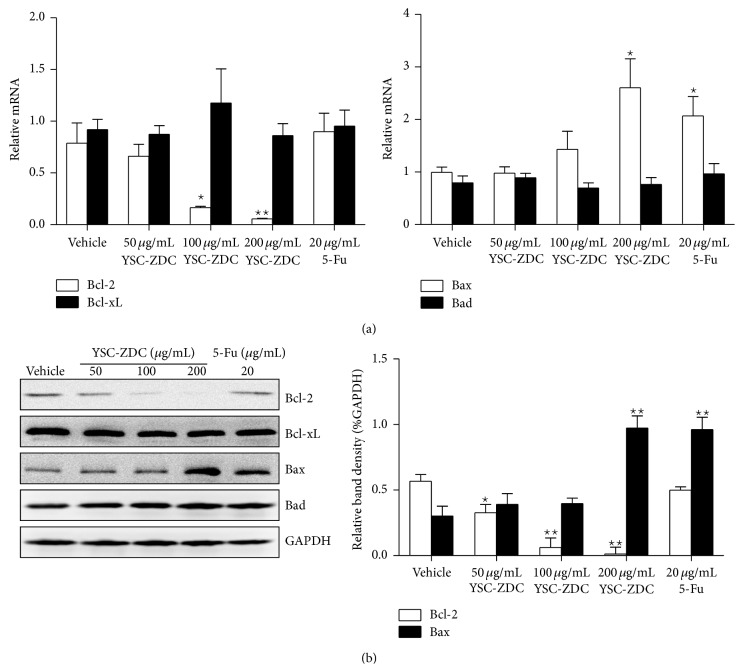
The effect of YSC-ZDC on Bcl-2 family proteins expression in Hep3B cells. (a) Cells were treated with YSC-ZDC at different concentrations or 20 *μ*g/mL 5-Fu for 24 h. Total RNA from Hep3B was isolated and mRNA levels of Bcl-2 family protein were determined by qPCR. (b) Cells were treated with YSC-ZDC at different concentrations or 20 *μ*g/mL 5-Fu for 48 h. The levels of Bcl-2, Bcl-xL, Bax, and Bad were determined by western blot. Bands were analyzed by densitometry. GAPDH was used as the endogenous control. Data are shown as means ± S.E.M. ^*^ *P* < 0.05, ^**^ *P* < 0.01 versus vehicle.

Bcl-2 family proteins serve as critical regulators of pathways involved in apoptosis, acting to either inhibit or promote cell death [13]. Next, we examined the effect of YSC-ZDC on Bcl-2 family proteins expression. Hep3B cells were treated with YSC-ZDC at different concentrations for 12 h, total RNA was isolated, and Bcl-2 family proteins mRNA expression was determined by real-time PCR. As shown in Figure 5(a), YSC-ZDC decreased Bcl-2 mRNA level and increased Bax mRNA level in a dose-dependent manner. But YSC-ZDC has no effect on Bcl-xL and Bad mRNA expression. As a positive drug, 5-Fu induced Bax mRNA expression. Consistent with PCR data, western blot assay showed that YSC-ZDC decreased Bcl-2 protein expression and increased Bax protein expression in a dose-dependent manner (Figure 5(b)).

### 3.6. YSC-ZDC Inhibited Growth Hepatoma Xenografts* In Vivo*

**Figure 6:**
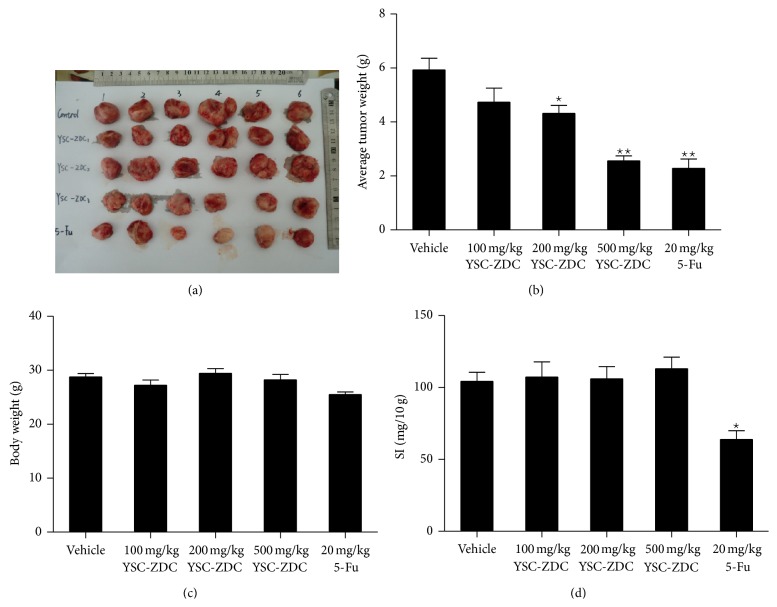
YSC-ZDC inhibited growth hepatoma xenografts* in vivo.* Hep3B cells (10^6^cells/200 *μ*L) were subcutaneously injected into the left flank of the mice. Indicated doses of YSC-ZDC were administered via gavage daily for 20 days. 5-FU was dissolved in 0.9% normal saline and administered intraperitoneally (20 mg/kg/day) for 2-day intervals as a positive control group. After 20 days, the mice were sacrificed and Hep3B xenograft tumors were excised and photographed. (a) Tumor volumes. (b) Tumor weight. (c) Body weight. (d) The spleen index (SI, spleen/body weight). Data are shown as means ± S.E.M. ^*^ *P* < 0.05, ^**^ *P* < 0.01 versus vehicle.

To investigate effects of YSC-ZDC* in vivo*, we subcutaneously injected 1 × 10^6^ Hep3B cells into the left parts of athymic nude mice. After all mice formed tumors, different doses of YSC-ZDC were administered via gavage daily for 20 days. As shown in Figures 6(a) and 6(b), YSC-ZDC at the doses of 200 and 500 mg/kg significantly inhibited weights of tumor xenografts. 20 mg/kg 5-Fu, as a positive control, also significantly inhibited tumor weight. YSC-ZDC administration has little impact on body weight of mice and the spleen index, whereas 5-FU-treated mice had much lower body weights and the spleen index than control group. These data indicated that YSC-ZDC significantly inhibited growth hepatoma xenografts* in vivo, *and unlike 5-FU, YSC-ZDC had little side effect on nude mice.

### 3.7. YSC-ZDC Inhibited Growth Hepatoma Xenografts by Regulating Bcl-2 Family Proteins Expression

**Figure 7:**
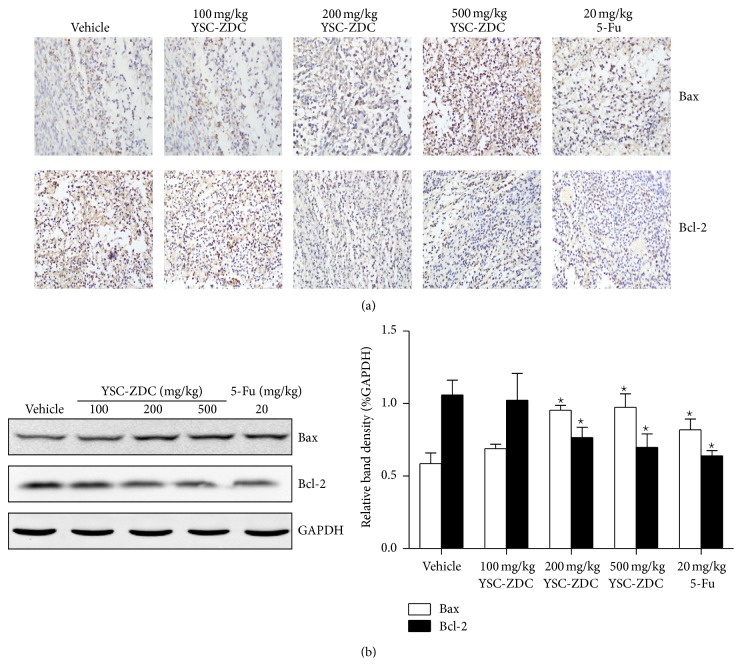
YSC-ZDC inhibited growth hepatoma xenografts by regulating Bcl-2 family protein expression. (a) Tumor tissues were fixed and Bcl-2 and Bax levels were determined by IHC. The images were visualized and analyzed using an inverted microscope. (b) The levels of Bcl-2 and Bax in tumor tissues were determined by western blot. Bands were analyzed by densitometry. GAPDH was used as the endogenous control. Data are shown as means ± S.E.M. ^*^ *P* < 0.05 versus vehicle.

Next, we determined the levels of Bcl-2 family proteins in tumor xenografts using IHC and western blot. The IHC result showed that administrating YSC-ZDC increased Bax protein level and decreased Bcl-2 protein level in tumor tissue (Figure 7(a)). Consistent with IHC result, western blot data showed that YSC-ZDC dose-dependently increased Bax protein level and decreased Bcl-2 protein level in tumor tissue (Figure 7(b)). These data indicated that YSC-ZDC exhibited antitumor activity through regulating Bcl-2 family proteins expression.

## 4. Discussion

Natural compounds isolated from medicinal plants, as rich sources of novel anticancer drugs, have shown over the years to have various biological activities. Traditional Chinese medicine has been used for pharmaceutical and dietary therapy for several millennia with more effective and fewer side effects [14]. In this study, we prepared the n-butanol extracts of* Pterocephalus hookeri* and investigated its antitumor activity. It is found that YSC-ZDC inhibited proliferation of cancer cell lines, induced apoptosis, and inhibited growth hepatoma xenografts through blocking of PI3K/Akt pathway and regulation of Bcl-2 family proteins expression.

Apoptosis is an active physiological process resulting in cellular self-destruction following specific morphological and biochemical changes in the nucleus and cytoplasm [15]. We found that YSC-ZDC exhibited proapoptotic activity as assessed by fluorescence microscopy, TEM, and flow cytometry. Blocking PI3K/Akt signaling pathway is potentially its underlying mechanism. Numerous studies have demonstrated that this pathway plays well-documented roles in carcinogenesis and drug resistance in HCC cells [16]. PI3K(s) are a family of enzymes involved in cellular functions such as cell growth, proliferation, differentiation, motility, survival, and intracellular trafficking, which in turn are involved in cancer [17]. As a downstream protein, PDK1 is phosphorylated and activated by PI3K and in turn active Akt which regulates cell survival and proliferation and inhibits apoptosis [18]. Our results revealed that YSC-ZDC significantly inhibited PDK1 phosphorylation in a dose-dependent manner, but YSC-ZDC has no effect on PI3K phosphorylation (Figure 4). The results indicated that YSC-ZDC could interrupt the interaction between PI3K and PKD1. This observation also suggests that blocking interaction of PI3K and PDK1 may be a target of medicinal plants in the treatment of cancer.

Hepatocellular carcinoma (HCC) derived from hepatocytes is one of the most common malignancies throughout the world. It is characterized by its high incidence in hepatitis B virus-associated cirrhotic liver disease [19] and other risk factors such as hepatitis C virus, aflatoxin, sex, hormones, and some metabolic diseases [20]. Impaired apoptosis is both critical in HCC development and a major barrier to effective treatment of HCC [21]. One mechanism by which Akt prevents apoptosis is considered to proceed through phosphorylation of the proapoptotic protein Bad on Ser-136 [22]. In addition, downregulation of Bcl-2 expression has been identified as a critical mechanism for cancer therapy. It has been reported that Akt upregulates Bcl-2 expression whose promoter region contains a cAMP-response element (CRE) site [23–25]. Our real-time PCR and western blot assay showed that YSC-ZDC inhibited Bcl-2 protein expression and increased Bax protein expression (Figure 5). Also YSC-ZDC administration inhibited growth hepatoma xenografts by regulating Bcl-2 family proteins expression (Figure 7). In line with previous report, we found that 5-Fu regulated Bcl-2 family proteins expression* in vivo*. Interestingly, 5-Fu increased Bax expression and failed to inhibit Bcl-2 protein expression in Hep3B cells. As a pyrimidine analog, 5-Fu causes the DNA strand to break and eventually leads to apoptosis in the cancer cell. 5-Fu may indirectly regulate Bcl-2 family expression due to inducing apoptosis. Our results suggested that YSC-ZDC might regulate Bcl-2 family proteins expression through blocking PI3K/Akt signaling pathway. It was reported that Akt regulates cell survival and apoptosis by inhibiting Bax conformational change [26]. As n-butanol extracts of* Pterocephalus hookeri*, YSC-ZDC exhibited its multitarget and minimal side effects. Further study about the antitumor activity of YSC on the cross talk of PI3K/Akt and Bcl-2 family proteins should be done.

Traditional Chinese medicine (TCM) such as Tibetan herbs has a long history. Although TCM-derived drugs were shown to be useful in clinical studies, there is a clear caveat with regard to the safety of compounds derived from TCM [27, 28]. Our study reported that unlike 5-Fu, YSC-ZDC exhibited lower side effect without effects on body weight and spleen index (Figure 6). Cancer prevention and treatment using traditional Chinese medicines have attracted increasing interest. However, their chemical and pharmacological bases are not well understood in most cases [29, 30]. As a first-line drug, Sorafenib improves survival in patients with hepatocellular carcinoma through inhibiting the serine/threonine kinases and the receptor tyrosine kinase activity of vascular endothelial growth factor receptors (VEGFRs). The antitumor activity of YSC on inhibiting these kinases activities should be further investigated. Increased knowledge of the molecular mechanisms of TCM-derived drugs will provide an attractive strategy for the development of novel and improved cancer therapeutics.

## 5. Conclusion

The results obtained in this study suggested that n-butanol extracts of* Pterocephalus hookeri *(YSC-ZDC) treatment inhibited cell proliferation, induced apoptosis, blocked PI3K pathway, and regulated levels of Bcl-2 family proteins* in vitro*. Administrating YSC-ZDC inhibited growth hepatoma xenografts by regulating Bcl-2 family proteins expression* in vivo*. These data demonstrated that YSC-ZDC treatment may be an effective therapeutic strategy for cancer.

## Figures and Tables

**Table 1:** IC50 values of YSC-ZDC in various tumor cell lines* in vitro*.

Cell type	Tumor type	IC50 (*μ*g/mL)
Hep3B	Hepatoma	90.8
ECA-109	Esophageal carcinoma	143.2
Caco-2	Colorectal adenocarcinoma	175.2
Hela	Cervix epithelioid carcinoma	203.8
K562	Chronic myelogenous leukemia	102.2
MCF-7	Breast adenocarcinoma	318.2
A549	Lung carcinoma	229.2

Cells were treated with various concentrations of YSC-ZDC for 48 h. The IC50 values shown were means ± S.E.M.
